# Improved longevity and *in vivo* performance of neurotransmitter detection using 30 µm cone-shaped carbon fiber microelectrode

**DOI:** 10.3389/fbioe.2025.1579380

**Published:** 2025-08-22

**Authors:** Haeun Kwon, Hyun-U Cho, Jeongeun Sim, Kyung-Jun Boo, Yumin Kang, Sangmun Hwang, Youngjong Kwak, Jaehyun Jang, Kyung Min Kim, Se Jin Jeon, Chan Young Shin, Kevin E. Bennet, Yoonbae Oh, Hojin Shin, Kendall H. Lee, Dong Pyo Jang

**Affiliations:** ^1^ Department of Electronic Engineering, Hanyang University, Seoul, Republic of Korea; ^2^ Department of Biomedical Engineering, Hanyang University, Seoul, Republic of Korea; ^3^ School of Medicine and Center for Neuroscience Research, Konkuk University, Seoul, Republic of Korea; ^4^ Department of Pharmacology, College of Medicine, Hallym University, Chuncheon, Republic of Korea; ^5^ SOSO H&C Co., Seoul, Republic of Korea; ^6^ Department of Healthcare Digital Engineering, Hanyang University, Seoul, Republic of Korea; ^7^ Department of Neurologic Surgery, Mayo Clinic, Rochester, MN, United States; ^8^ Division of Engineering, Mayo Clinic, Rochester, MN, United States; ^9^ Department of Biomedical Engineering, Mayo Clinic, Rochester, MN, United States

**Keywords:** Electrochemistry, carbon fiber microelectrode, electrochemical etching, dopamine, longevity, tissue damage

## Abstract

Fast Scan Cyclic Voltammetry (FSCV) is a widely used electrochemical technique to detect rapid extracellular dopamine transients *in vivo*. It employs carbon fiber microelectrodes (CFMEs), but conventional 7 µm diameter CFMEs often suffer from limited mechanical durability and reduced lifespan, hindering their use in chronic monitoring. To improve mechanical robustness and long-term functionality, we fabricated 30 µm diameter CFMEs and modified their geometry via electrochemical etching to form cone-shaped tips. We compared the *in vitro* and in vivo performance of 7 µm, 30 µm bare, and 30 µm cone-shaped CFMEs using FSCV. Electrode longevity was assessed, and biocompatibility was evaluated via immunofluorescence analysis of brain tissue. *In vitro*, the 30 µm bare CFMEs showed 2.7-fold higher sensitivity (33.3 ± 5.9 pA/µm^2^, n = 5) compared to 7 µm CFMEs (12.2 ± 4.9 pA/µm^2^, n = 5). However, in vivo dopamine detection was significantly reduced in 30 µm bare CFMEs (12.9 ± 8.1 nA, n = 5) relative to 7 µm CFMEs (24.6 ± 8.5 nA, n = 5), likely due to tissue damage. Cone-shaped modification of 30 µm CFMEs resulted in a 3.7-fold improvement in vivo dopamine signals (47.5 ± 19.8 nA, n = 5) and significantly lower glial activation based on Iba1 and GFAP markers. Furthermore, erosion tests revealed a 4.7-fold increase in lifespan compared to 7 µm CFMEs. These results suggest that while increasing CFME diameter improves sensitivity, it also increases tissue damage *in vivo*. The cone-shaped geometry effectively mitigates insertion-induced damage, enhancing *in vivo* performance and biocompatibility. This design offers a promising approach for long-term neurotransmitter monitoring and potential integration into closed-loop neuromodulation systems.

## Introduction

Fast Scan Cyclic Voltammetry (FSCV) is a well-established technique to detect rapid transients in extracellular dopamine levels. Carbon fiber microelectrodes (CFMEs) are particularly suitable for detecting catecholamines due to the oxide functional groups on their surfaces enhancing cation adsorption (Venton and Cao, 2020). Standard CFMEs, typically 7–10 µm in diameter (Smith et al., 2018; Nicolai et al., 2018; Hensley et al., 2018; Pitts et al., 2020), offer high conductivity (Yang et al., 2015; Chatard et al., 2018) and excellent biocompatibility. Their small diameter minimizes tissue damage, as they are comparable in size to neurons (Roberts et al., 2013; Hejazi et al., 2020). In addition, FSCV waveform repetition enables partial electrochemical surface renewal at the CFME interface, helping sustain sensitivity by mitigating surface fouling during chronic use (Takmakov et al., 2010). These features, combined with the temporal and spatial resolution provided by FSCV, make CFMEs highly effective for detecting catecholamines (Venton and Cao, 2020; Robinson et al., 2003).

The advantages of FSCV have led to its widespread use in neuroscience research. Studies using awake animal models have explored the relationship between animal behavior and phasic neurotransmitter release (Shnitko et al., 2017; Stelly et al., 2020; Schluter et al., 2014). Moreover, *in vivo* dopamine recording has been increasingly employed to study chronic neurochemical dynamics in animal models (Clark et al., 2010; Oh et al., 2016; Schwerdt et al., 2017; Howe et al., 2013). Since dopamine plays a critical role in brain functions and is implicated in neurological disorders, including Parkinson’s disease, monitoring extracellular neurotransmitter concentration over time is essential for understanding these conditions and developing treatments (Zhou et al., 2001; Piccart et al., 2015).

Beyond animal models, FSCV has been used to investigate the connection between neurotransmitters and human behavior (Kishida et al., 2016; Bang et al., 2020). For instance, Kishida et al. (2016) measured dopamine levels in individuals with Parkinson’s disease during decision-making tasks. They found that dopamine fluctuations both reward prediction and counterfactual error terms. Similarly, FSCV has been employed to study the effects of deep brain stimulation (DBS), a treatment for neuropsychiatric disorders (Chang et al., 2012). Chang et al. (2012) demonstrated that DBS electrodes induced the release of neurochemicals, including adenosine. These findings underscore FSCV’s potential to provide real-time feedback for closed-loop DBS systems, which provide adaptive stimulation based on neurotransmitter levels to optimize therapeutic outcomes. Despite this promise, the chronic use of FSCV is limited by the over-oxidation of carbon fibers, which leads to mechanical degradation and diminished electrode performance (Kozai et al., 2015; Patel et al., 2020; Takmakov et al., 2010; Bennet et al., 2016).

To address these limitations, several approaches can be suggested to enhance the durability and longevity of CFMEs in FSCV. Metal electrodes, such as gold or platinum, offer mechanical robustness, reducing the risk of breakage during insertion (Zachek et al., 2008; Zhao and Liu, 2018; Mattioli et al., 2019; Adams et al., 2020; Li et al., 2022). However, chronic implantation is limited by passivation and biofouling, which may trigger immune responses and damage surrounding tissue (Patrick et al., 2011; Roberts and Sombers, 2018; Lucio Boschen et al., 2021). Surface coatings, such as boron-doping or Nafion, can improve stability and sensitivity but may compromise temporal resolution and degrade with repeated use (Zanin et al., 2014; Kalaiyarasi et al., 2017; Wilson et al., 2018; Lucio Boschen et al., 2021). Lastly, increasing electrode diameter can improve mechanical strength and sensitivity, as shown by studies on 32 µm CFMEs (Chadchankar and Yavich, 2012). While this study highlights the potential of using larger-diameter electrodes in FSCV, there is limited evidence evaluating their long-term durability and suitability for *in vivo* applications.

This study addresses these limitations by comparing the performance of 30 μm and 7 µm CFMEs in both *in vitro* and *in vivo*. To minimize tissue damage and improve biocompatibility, the 30 µm CFMEs were further modified into a cone shape using electrochemical etching. Immunofluorescence analysis was conducted to assess tissue responses to electrode insertion, and an erosion test was performed to evaluate electrode durability. The results demonstrated that 30 µm cone-shaped CFMEs provide superior sensitivity, biocompatibility, and longevity, supporting their potential for chronic dopamine monitoring and closed-loop DBS applications.

## Materials and methods

### Fabrication of carbon fiber microelectrodes

CFMEs were fabricated following established protocols (Oh et al., 2016). AS4 carbon fiber (Hexcel, Stamford, CT) was used for 7 µm CFMEs, and 30 µm carbon fiber was obtained from World Precision Instruments (WPI, FL, United States). The exposed carbon fiber was trimmed to a final length of approximately 100 µm using a scalpel. All CFMEs were preconditioned before dopamine detection using a 1.5 V FSCV sweep (−0.4–1.5 V at 400 V/s, 30 Hz) and a standard FSCV waveform (−0.4–1.3 V sweep; 10 Hz).

### Electrochemical etching of 30 µm carbon fiber microelectrodes

30 µm cone-shaped CFMEs were fabricated using a homemade electrochemical etching system, as shown in Figure 2a. Based on the protocol by Hobara et al. (2007), a direct current voltage of 10 V was applied to a 1 mm segment of carbon fiber submerged in Tris buffer (Hobara et al., 2007). The carbon fiber underwent electrolysis for 20 s, resulting in partial erosion and detachment. A linear actuator moved the electrode upward at a constant speed during etching, gradually exposing it to air and forming the desired cone shape. The final cone height was controlled between 100 and 120 µm by adjusting the actuator speed.

### Chemicals

Tris buffer was used to dissolve and dilute the dopamine stock solution. Although artificial cerebrospinal fluid more closely mimics *in vivo* conditions, Tris buffer was chosen to ensure electrochemical stability and signal consistency in controlled *in vitro* conditions. The buffer pH was adjusted to 7.4 using a mixture of 15 mM Trizma phosphate, 3.25 mM KCl, 140 mM NaCl, 1.2 mM CaCl_2_, 1.25 mM NaH_2_PO_4_, 1.2 mM MgCl_2_, and 2.0 mM Na_2_SO_4_. All chemicals were purchased from Sigma-Aldrich (St. Louis, MO, United States). Dopamine was dissolved in Tris buffer to a stock concentration of 1 mM and stored with 50 µM perchloric acid. The stock solution was further diluted in Tris buffer to prepare experimental concentrations.

### Data acquisition and analysis

All experiments, except for the longevity test, were conducted using an FSCV waveform of −0.4–1.3 V sweep at 10 Hz. Data were acquired using a commercial electrical interface (NI USB-6363, 16-bit, National Instruments) connected to a base station PC with custom LabVIEW 2016 software (National Instruments, Austin, TX). The recorded data, stored as sequences of unsigned 2-byte integers, were processed using MATLAB (MathWorks Inc., Natick, MA), which applied filtering and background subtraction. The signal-to-noise ratio (SNR) was calculated by dividing the signal amplitude for 1 µM dopamine by the average noise amplitude, measured over a 10 s window preceding the dopamine addition by 3 s. Statistical analyses and figure generation were conducted using GraphPad Prism 10 (GraphPad Software, San Diego, CA). Normality and variance assumptions were assessed using the Shapiro-Wilk test and F-test, and appropriate statistical tests were selected accordingly. Exact p-values and confidence intervals (CI) are provided in the main text where relevant. Confidence intervals were calculated for comparisons with multiple replicates (n ≥ 3), all reported CIs represent 95% confidence. All results are reported as mean ± standard deviation (SD) for n electrodes or rats.

The longevity of CFMEs was evaluated using the in-house Voltammetry Instrument for Neurochemical Applications (VINA) system, which enables simultaneous testing of multiple electrodes with customizable waveforms (Marsh et al., 2017). FSCV waveforms (−0.4–1.5 V sweep; 30 Hz) were applied continuously, and electrode conditions were periodically inspected using voltammogram and microscopy. Electrodes were monitored until complete loss of background current was observed, which was defined as the failure point.

### Animal preparation and surgical procedures

Adult male Sprague-Dawley rats (Orient Bio, South Korea) were used for *in vivo* studies. All animal experiments were approved by Hanyang University’s Institutional Animal Care and Use Committee (IACUC, Seoul, South Korea). Rats were housed under a 12-h light/dark cycle with controlled temperature and humidity and given *ad libitum* access to food and water. Anesthesia was induced via intraperitoneal injection (i.p.) of urethane (1.6 g/kg, Sigma-Aldrich, St. Louis, MO), and surgeries were performed using a stereotaxic frame (Model 900, David Kopf Instruments, Tujunga, CA).

Working, stimulating, and reference electrodes were surgically implanted to evoke and detect phasic dopamine. Stimulating electrodes (Plastic One, MS303/2, Roanoke, VA, United States) were unilaterally placed in the medial forebrain bundle (coordinates: AP −4.6 mm; ML +1.4 mm; DV -8.5 to −9.0 mm). Ag/AgCl reference electrodes were placed in superficial cortical tissue. The CFME working electrode was inserted in the striatum (coordinates: AP +1.2 mm; ML +2.0 mm; DV -4.5 to −6.0 mm). Electrical stimulation was applied as bipolar pulse trains (2 m pulse width, 300 μA, 60 Hz) for 2 s every 10 min using the WINCS Harmoni system (Lee et al., 2017).

### Scanning electron microscopy/energy-dispersive X-ray spectroscopy (SEM/EDS)

SEM imaging was performed using a MIRA3 system (TESCAN, Brno, Czech Republic) to analyze the electrode tip morphology. A platinum coating was applied to prevent electron charging. Imaging parameters included an acceleration voltage of 15 kV. EDS was used to determine the chemical composition of the electrode surface.

### Immunofluorescence

Immunofluorescence analysis was conducted to investigate immune activation. Either 30 µm bare or cone-shaped CFMEs were implanted in the rat striatum. After 21 days, anesthetized rats were perfused transcardially with 4% paraformaldehyde (PFA, BIOSESANG, Republic of Korea). Brains were extracted and post-fixed in 4% PFA for 24 h and cryoprotected in 30% sucrose for 3 days. Frozen brain sections (30 µm thick) were prepared using a cryotome (Leica CM1520) and stained with primary antibodies against Iba1 (Ab. Cam AB5076, 1:200) and GFAP (Abcam AB7260, 1:200). Secondary antibodies, Alexa Fluor 546 (Invitrogen A10036, 1:250) and Alexa Fluor 488 (Invitrogen A11055, 1:250), were applied, followed by DAPI counterstaining. Sections were mounted using Prolong Gold antifade reagent (Invitrogen P36930) and imaged using a confocal laser scanning microscope (Carl Zeiss LSM900).

Immune response analysis was conducted using ImageJ software. The insertion site was defined as the region of interest (ROI, the yellow box in Figure 5). The number and integrated density of Iba1-and GFAP-positive cells were compared to contralateral, non-implanted brain regions. Quantitative data were normalized to the non-inserted control hemisphere.

## Results

### Comparative analysis of 7 μm and 30 µm bare carbon fiber microelectrodes: *In vitro* and *in vivo*

**FIGURE 1:**
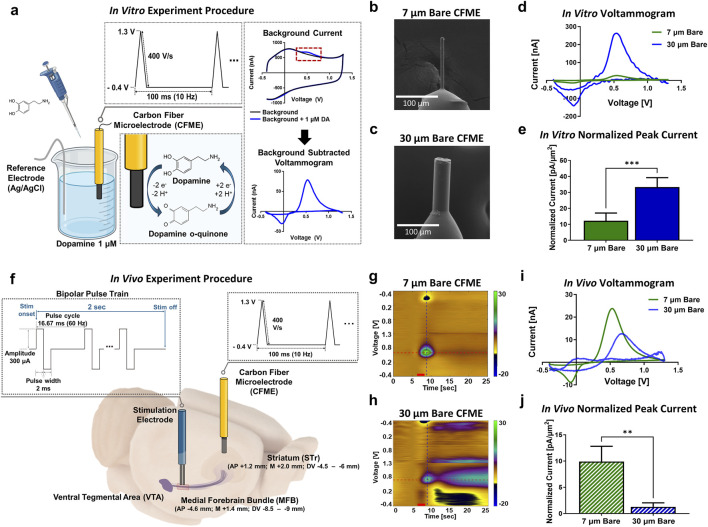
*In vitro* and *in vivo* properties of 7 μm and 30 µm bare CFMEs. **(a)** FSCV procedure for measuring 1 µM dopamine in a beaker **(b,c)** SEM images of 7 μm and 30 µm diameter CFME. **(d)** Voltammograms for dopamine detection in 1 µM dopamine solution. **(e)** Mean peak oxidation currents, normalized to electrode surface area. **(f)** FSCV procedure for measuring evoked phasic dopamine release *in vivo* **(g,h)** FSCV color plots showing phasic dopamine responses in the striatum recorded by 7 μm and 30 µm CFMEs **(i)** *In vivo* voltammograms for dopamine detection in the striatum. **(j)** Mean peak oxidation currents, normalized to electrode surface area. Green and blue color represent 7 μm and 30 µm bare CFMEs, respectively. Data are presented as mean ± SD. ***p < 0.001, **p < 0.01.

To evaluate the performance of 7 μm and 30 µm bare CFMEs, background current, sensitivity, and signal-to-noise ratio (SNR) were measured during *in vitro* dopamine detection. Dopamine (1 µM) was introduced into a beaker, and a standard FSCV waveform (−0.4 to 1.3 V, 10 Hz) was applied, as illustrated in Figure 1a. Both CFMEs were fabricated using identical methods and trimmed to approximately 100 µm in length, as shown in Figures 1b,c. The 30 µm bare CFMEs exhibited an 11.0 times higher peak oxidation current (300.6 ± 86.8 nA, n = 5) compared to the 7 µm CFMEs(27.3 ± 10.9 nA, n = 5) (Figure 1d). To compare sensitivity, the oxidation peak currents were normalized to the electrode surface area. The normalized sensitivity of 30 µm bare CFMEs (33.3 ± 5.9 pA/μm^2^, n = 5) was 2.7 times higher than that of 7 µm CFMEs (12.2 ± 4.9 pA/μm^2^, n = 5; unpaired Student’s t-test, p = 0.0003, 95% CI [13.18, 28.97]) (Figure 1e). This difference may reflect variations in the physical properties of the carbon fibers, potentially influenced by multiple factors, including manufacturing process. The SNR was computed as the ratio between the signal amplitude for 1 µM dopamine and the average noise amplitude measured over 10 s before dopamine addition. 30 μm bare CFMEs showed significantly higher SNR (96.1 ± 1.4 dB, n = 5) than to the 7 µm CFMEs (60.4 ± 1.4 dB, n = 5; Welch’s t-test, p < 0.0001, 95% CI [30.37, 40.97]) (Supplementary Figure S1).

To assess *in vivo* dopamine detection capability, we stimulated the MFB and recorded dopamine levels in the hot spots of the striatum, targeting areas with the highest neurotransmitter release (Figure 1f). Contrary to the *in vitro* results, the 30 µm bare CFMEs showed lower *in vivo* dopamine detection signals (12.9 ± 8.1 nA, n = 5) compared to the 7 µm CFMEs (24.6 ± 8.5 nA, n = 5), despite their higher *in vitro* sensitivity. When normalized to electrode surface area, the 30 µm bare CFMEs (1.3 ± 0.8 pA/μm^2^, n = 5) achieved only 13.1% of the normalized current of 7 µm CFMEs (9.9 ± 2.9 pA/μm^2^, n = 5; Welch’s t-test, p = 0.0019, 95% CI: [−12.18, −5.08]) (Figure 1j). This discrepancy is likely due to tissue damage caused by the insertion of the thicker and blunter 30 µm electrodes, which can interfere with local neurotransmitter release at the recording site (Jaquins-Gerstl and Michael, 2009; Bungay et al., 2003).

### *In vitro* properties of 30 µm cone-shaped carbon fiber microelectrodes

**FIGURE 2:**
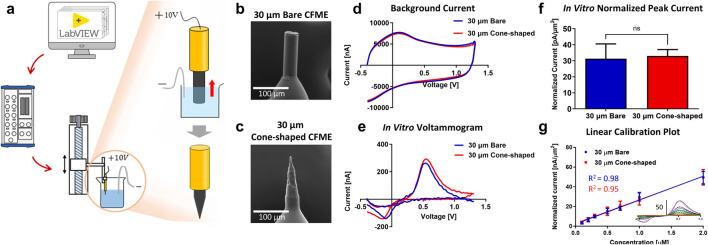
*In vitro* properties of 30 µm bare and cone-shaped CFMEs. **(a)** Schematic of the homemade electrochemical etching system **(b,c)** SEM images of 30 µm bare and cone-shaped CFME. **(d,e)** Background current and dopamine detection voltammograms in 1 µM dopamine solution. **(f)** Mean peak oxidation currents (n = 8), normalized to electrode surface area. **(g)** Dopamine detection ranges from 100 nM to 2 µM (n = 3) and linear correlation (*R* ^2^) between dopamine concentration and peak oxidative current. In the voltammograms, solid lines represent the 30 µm bare electrodes, while dashed lines indicate the 30 µm cone-shaped electrodes. Currents were normalized to electrode surface area. Blue and red color represent 30 µm bare cone-shaped CFMEs, respectively. Data are presented as mean ± SD. ns, non-significant.

To reduce tissue damage caused by electrode insertion, the tip of the 30 µm bare CFMEs was modified into a sharp, conical geometry using an electrochemical etching process (Figure 2a). During etching, the carbon fiber was eroded by electrolysis, transforming the cylindrical tip into a cone shape as the electrode was gradually withdrawn from the electrolyte (Figure 2b). The final cone geometry, shown in Figure 2c, was achieved by modulating the actuator speed to control the cone height between 100 and 120 µm. EDS analysis confirmed that the surface composition of the carbon fiber remained unchanged before and after the etching (Supplementary Figure S2). Electrochemical properties showed that 30 µm bare and cone-shaped CFMEs exhibited similar background and faradaic currents for dopamine detection (Figures 2d,e). Analysis of normalized peak currents revealed no significant difference in sensitivity between the two designs (n = 8; Welch’s t-test, p = 0.6494, 95% CI: [−6.29, 9.62]) (Figure 2f).

Electrode sensitivity was evaluated by measuring current responses across a range of dopamine concentrations, from 100 nM to 50 µM. For each concentration, dopamine was added to a beaker containing Tris buffer and stirred, following the same procedure described in Figure 1a. Analysis was performed using the plateau phase of the current response following the dopamine-induced increase. Dopamine response curves across concentrations from 100 nM to 2 µM also showed consistent sensitivity between electrode types, with comparable slopes of normalized current versus concentration (24.2 and 23.8, respectively) (n = 3 per group; Two-way ANOVA, p = 0.7551, 95% CI: [−3.12, 2.29]) (Figure 2g). Both 30 µm bare and cone-shaped CFMEs exhibited diffusion-controlled kinetics, as evidenced by current saturation due to adsorbed dopamine. Two-way ANOVA revealed no significant difference in sensitivity between the two electrode types (n = 3 per group; p = 0.7882, 95% CI [-12.43, 9.50]) (Supplementary Figure S3).

These results indicate that electrochemical etching preserved the intrinsic electrochemical properties of the 30 µm CFMEs, while primarily improving its physical geometry for insertion without compromising detection sensitivity.

### Enhanced *in vivo* targeting of dopamine hot spots using 30 µm cone-shaped carbon fiber microelectrodes

**FIGURE 3:**
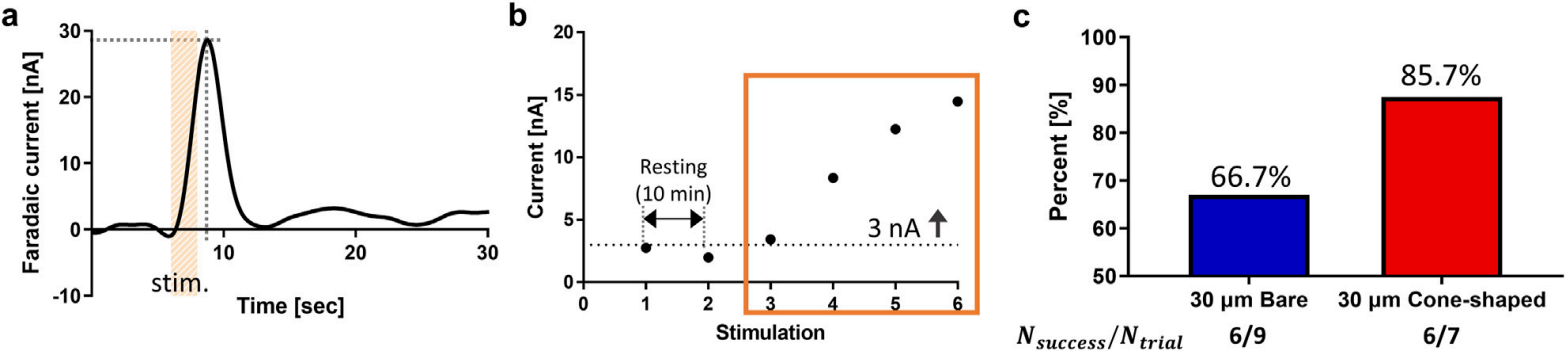
Targeting criteria and success rates for dopamine hot spots. **(a)** First criterion is rise in current at stimulation onset followed by a decline at offset, indicating a dopamine oxidation peak. **(b)** Second criterion is a minimum of 3 nA current for at least three consecutive stimulations. **(c)** Success rate of targeting striatal regions showing high dopamine activity, evaluated based on criteria **(a,b)** during CFME insertion. Success was achieved in 6 out of 9 attempts with 30 µm bare CFMEs and 6 out of 7 attempts with 30 µm cone-shaped CFMEs. All procedures were performed by an experienced technician.

Accurate targeting of dopamine-rich “hot spots”, defined as regions with a high density of neurotransmitter release, is essential for achieving reliable neurochemical measurements *in vivo*. However, the lower dopamine signals observed *in vivo* suggest that 30 µm bare CFMEs may have limited accuracy in targeting dopamine hot spots (Figure 1j). To investigate this, the efficacy of hot spot targeting in the rat striatum was compared between 30 µm bare and cone-shaped CFMEs. Targeting success was defined as dopamine release exceeding three standard deviations above baseline noise during FSCV, based on the average response to MFB stimulation (Figures 3a,b). Reliable detection of these hot spots depends on electrode sensitivity and minimizing tissue damage during insertion.

The 30 µm cone-shaped CFMEs achieved a success rate of 85.7% (6 out of 7 electrodes), compared to 66.6% (6 out of 9 electrodes) for the 30 µm bare CFMEs. This 19.1%p improvement highlights the advantage of the conical design in reducing insertion-related tissue disruption, which can otherwise impair neurotransmitter release even when electrodes are correctly positioned. The sharper geometry of the 30 µm cone-shaped CFMEs likely enables smoother tissue penetration, mitigating the mechanical stress and cellular disruption caused by blunt-tip insertion.

**FIGURE 4:**
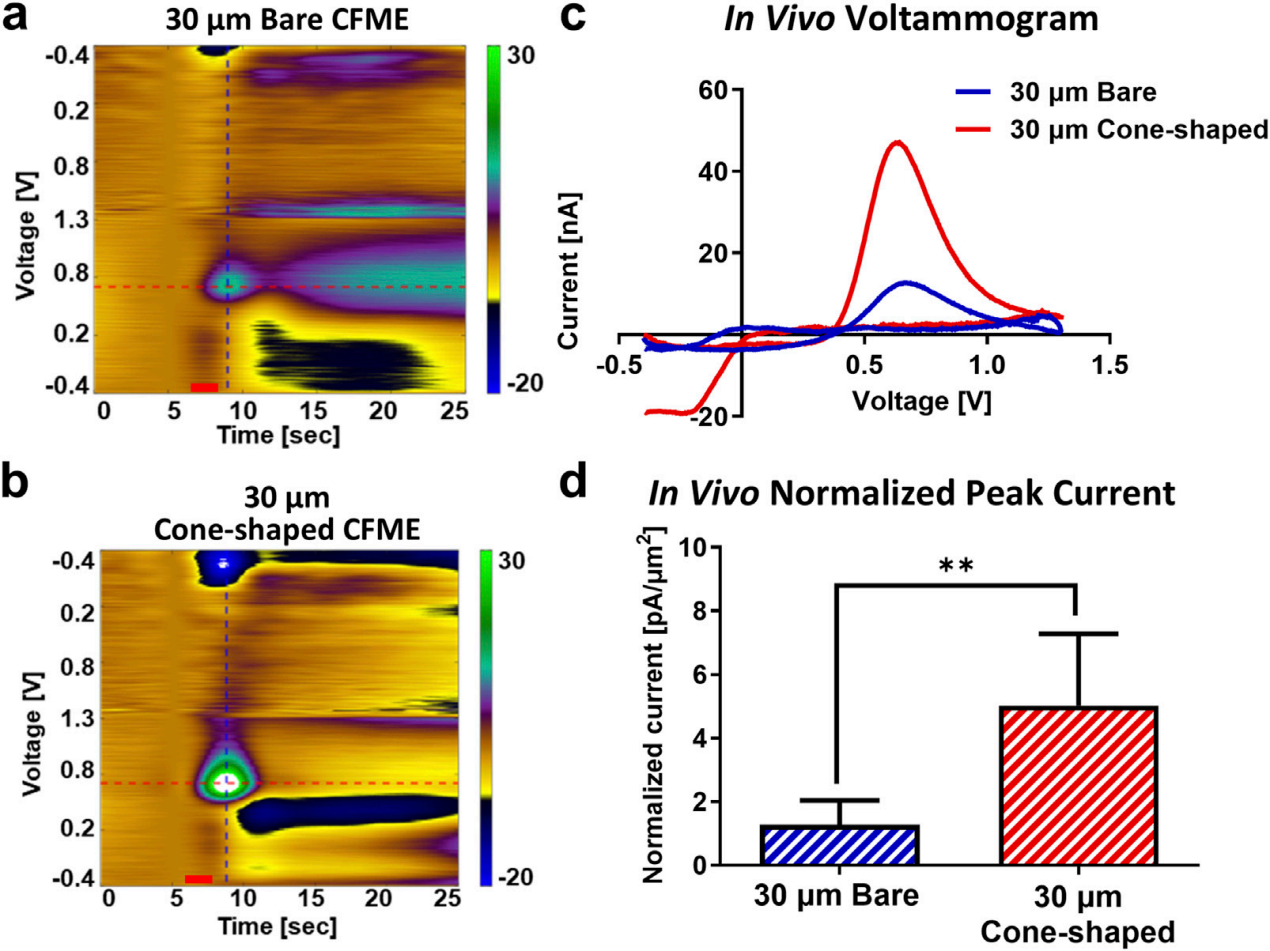
*In vivo* dopamine detection using 30 µm bare and cone-shaped CFMEs. **(a,b)** FSCV color plots showing phasic dopamine responses in the striatum. **(c)** *In vivo* voltammograms for dopamine detection. **(d)** Mean peak oxidation currents, normalized to electrode surface area. Blue and red color represents 30 µm bare and cone-shaped CFMEs, respectively. Data are presented as mean ± SD. **p < 0.01.

Quantitative analysis of faradaic currents in the striatum revealed significant improvements with the 30 µm cone-shaped CFMEs. Dopamine oxidation currents were 3.7-fold higher with the 30 µm cone-shaped CFMEs (47.5 ± 19.8 nA, n = 5) compared to the 30 µm bare CFMEs (12.9 ± 8.1 nA, n = 5), despite similar background currents (Figures 4a–c; Supplementary Figure S4). When normalized to the surface area, the 30 µm cone-shaped CFMEs achieved currents of 5.0 ± 2.3 pA/μm^2^, which was 3.8 times higher than the 1.3 ± 0.8 pA/μm^2^ observed with 30 µm bare CFMEs (n = 5; unpaired Student’s t-test, p = 0.0077, 95% CI [1.299, 6.201]). These results suggest that the reduced tissue damage from cone-shaped design enhances *in vivo* dopamine detection.

**FIGURE 5:**
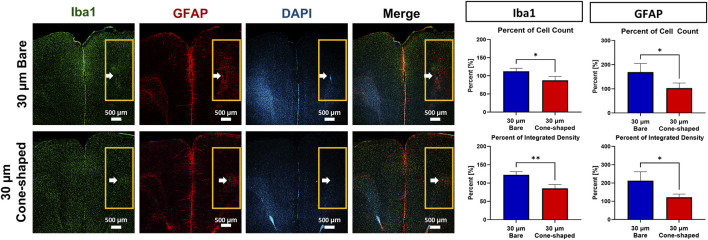
Immunofluorescence 21 days after implantation. A photograph of a rat brain after 30 µm bare CFME (n = 3) and 30 µm cone-shaped CFME (n = 4) removal. Coronal brain slices were stained with Iba1 (activated microglia), GFAP (astrocytes), and DAPI (nuclei). Fluorescent images show immune responses around the electrode site. Quantification of activated and integrated density of Iba1 and GFAP was performed using ImageJ within the ROI (yellow pox). Data are presented as mean ± SD.

Immunofluorescence was performed on brain tissues after 21 days implantation to assess the physiological impact of electrode insertion. The analysis focused on microglial and astrocytic activation using Iba1 and GFAP markers. The insertion site was designated as ROI, highlighted by the yellow box in Figure 5. The number and integrated density of Iba1-and GFAP-positive cells were compared with contralateral, non-inserted regions. Quantitative analysis revealed that the 30 µm cone-shaped CFMEs induced significantly lower immune responses than the 30 µm bare CFMEs.

Specifically, the 30 µm cone-shaped CFMEs exhibited significantly lower Iba1-positive cell counts (87.5% ± 10.6%) compared to the 30 µm bare CFMEs (112.4% ± 8.3%), indicating a marked reduction in microglial activation (unpaired Student’s t-test, p = 0.0203, 95% CI [−44.14, −5.82]). Similarly, the Iba1 integrated density was reduced by 36.8 percentage points in the cone-shaped group (85.4% ± 10.5%) relative to the bare group (122.2% ± 9.2%), showing a highly significant difference (p = 0.0047). A comparable trend was observed with GFAP staining. The 30 µm cone-shaped CFMEs elicited significantly lower GFAP-positive cell counts (102.8 ± 20.9) than the bare counterparts (169.1 ± 35.4; p = 0.0258, 95% CI [−120.60, −11.97]). GFAP integrated density also decreased substantially in the cone-shaped group (121.8 ± 17.4) compared to the bare group (212.5 ± 49.3; p = 0.0173, 95% CI [−157.4, −24.11]). This result demonstrates the high biocompatibility of the cone-shaped electrode.

### Extended lifetime of 30 µm cone-shaped carbon fiber microelectrodes

**FIGURE 6:**
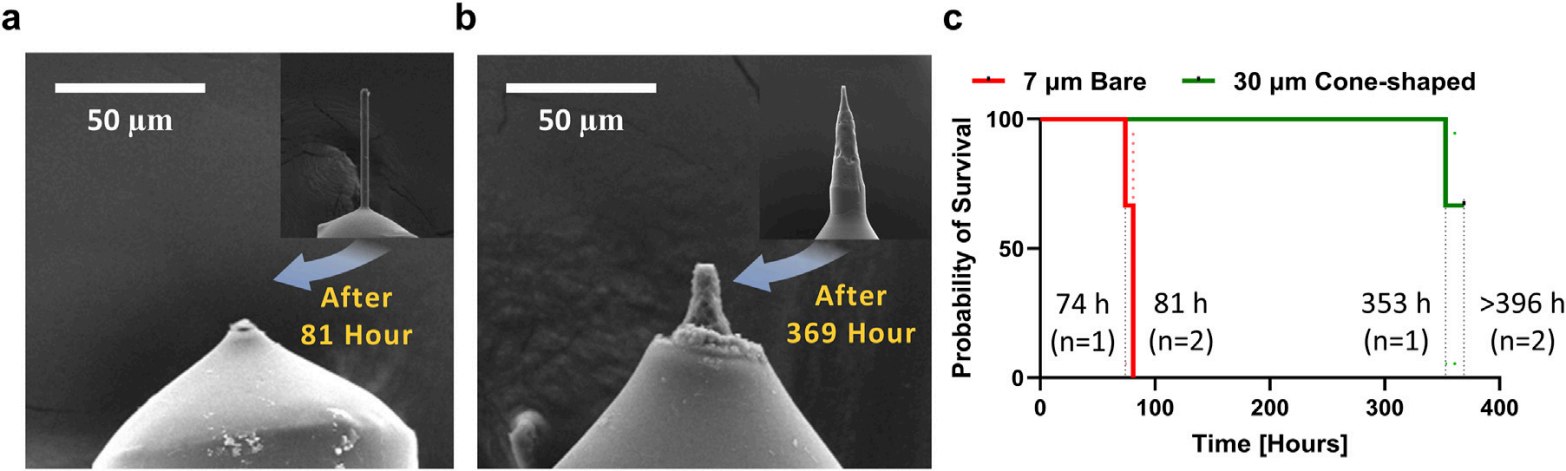
Long term electrochemical durability of 7 μm and 30 µm cone-shaped CFMEs. **(a,b)** SEM images of 7 μm and 30 µm cone-shaped CFMEs after continuous FSCV waveform application. Tip erosion was observed in 7 µm CFMEs, while 30 µm cone-shaped CFME tips remained. **(c)** Comparison of electrode survival probability between 7 μm and 30 µm cone-shaped CFMEs (n = 3)

Durability is an essential consideration for CFMEs in chronic *in vivo* applications. To evaluate electrode durability, we applied continuous FSCV waveforms (−0.4 to 1.5 V, 30 Hz) using the VINA system, enabling simultaneous testing of multiple electrodes under identical conditions (Marsh et al., 2017). The 7 µm CFMEs were fully eroded after an average of 78.7 ± 4.0 h (n = 3), with a complete loss of background current (Figure 6a). In contrast, the 30 µm cone-shaped CFMEs exhibited a markedly extended lifespan of 363.7 ± 9.2 h (n = 3). Two out of three electrodes retained background current beyond 369 h and were manually discontinued, as they did not reach a clear failure point during the test period. These were treated as right-censored data in the survival analysis. SEM imaging further confirmed the persistence of the carbon fiber tip, suggesting greater resistance to physical degradation (Figures 6b,c). The log-rank (Mantel-Cox) test indicated a significant difference between the two groups (p = 0.0295). The Mantel-Haenszel hazard ratio was 6.23 (95% CI [1.32, 199.6]), suggesting a substantially increased failure rate in the 7 µm CFMEs. On average, this difference corresponds to a 4.7-fold increase in electrode lifespan. These findings highlight the durability advantage of 30 µm cone-shaped CFMEs for chronic use.

## Discussion

This study presents notable improvements in dopamine detection using electrochemically etched 30 µm cone-shaped CFMEs. These electrodes demonstrated enhanced sensitivity and extended longevity compared to conventional 7 µm CFMEs. They also achieved higher accuracy *in vivo* hot spot targeting by alleviating tissue damage.

The 30 µm cone-shaped CFMEs exhibited significantly improved *in vitro* sensitivity. Peak currents increased by 9.9-fold, and normalized sensitivity was 2.7 times higher than 7 µm CFMEs. Previous studies have shown that electrode geometry, such as diameter, influences electrode sensitivity (Venton and Cao, 2020; Chadchankar and Yavich, 2012). The enhanced performance of the 30 µm cone-shaped CFMEs is likely attributed to their increased surface area, which facilitates dopamine adsorption without compromising response time, a common drawback observed in coated electrodes. The SNR improvement further supports their suitability for detecting low-concentration dopamine fluctuations, a critical requirement for capturing rapid neurochemical dynamics (Robinson et al., 2003).

Despite the improved performance observed *in vitro*, the 30 µm bare CFMEs did not exhibit superior sensitivity *in vivo*. The 30 µm cone-shaped CFMEs exhibited a current of 47.5 ± 19.8 nA in the striatum, representing a 1.9-fold increase over the 7 µm CFMEs. However, when normalized by the electrode surface area, the oxidation currents were 51% of the 7 µm CFMEs. This discrepancy may partially be due to residual cellular damage. A more likely explanation is the limited diffusion range of dopamine (5–10 µm), which prevents uniform interaction across the entire electrode surface (Cragg and Rice, 2004). As a result, the sensitivity normalized to surface area may appear reduced.

FSCV with 30 µm cone-shaped CFMEs significantly improved the detection of dopamine fluctuations *in vivo*, especially within dopamine-rich “hot spots” associated with behavior and learning. Prior studies have demonstrated that FSCV provides superior temporal resolution for capturing subsecond dopamine transients (Shnitko et al., 2017; Stelly et al., 2020; Schluter et al., 2014; Robinson et al., 2003). The increased sensitivity and stability of the 30 µm cone-shaped CFME make it well-suited for real-time monitoring of dopamine fluctuations. This is crucial for advancing the understanding of dopamine dynamics *in vivo*. These capabilities may support the development of personalized closed-loop DBS systems.

Our immunofluorescence analysis revealed reduced activation of Iba1 and GFAP around the insertion site of the 30 µm cone-shaped CFMEs compared to the 30 µm bare CFMEs. This suggests that the modified electrode geometry minimizes tissue disruption during insertion, potentially reducing neuroinflammation and improving chronic biocompatibility. These observations align with findings from McNamara et al. (2024), which showed that electro-sharpened electrodes induce less tissue damage than blunt electrodes. Given that neuroinflammation and gliosis negatively affect dopamine release and degrade signal quality (Kozai et al., 2015), the cone-shaped CFME design offers a critical advantage for sustained *in vivo* applications.

A key finding of this study is the extended lifespan of the 30 µm cone-shaped CFMEs, lasting approximately 4.7 times longer than the 7 µm CFMEs under continuous use. Survival analysis using the Mantel-Cox test revealed a significant difference in electrode longevity (p = 0.0295), with a hazard ratio of 6.23 (95% CI: 1.32–199.6), indicating a markedly increased failure risk in 7 µm CFMEs. This enhancement in durability is pivotal for chronic applications, addressing a major limitation of traditional CFMEs (Kozai et al., 2015; Patel et al., 2020; Takmakov et al., 2010; Bennet et al., 2016). The cone-shaped design’s improved longevity and surface renewal capability make it a promising candidate for long-term neurochemical monitoring and closed-loop DBS systems. Unlike coated or metallic electrodes, which are prone to passivation and degradation, the 30 µm cone-shaped CFME offers mechanical robustness and an extended operational lifespan (Patrick et al., 2011; Lucio Boschen et al., 2021; Chadchankar and Yavich, 2012).

While this study demonstrated the feasibility of using 30 µm cone-shaped CFMEs for chronic dopamine monitoring, several limitations remain. First, the immune response evaluation was limited to a 21-day period, and long-term signal stability was not assessed beyond this timeframe. Although the electrode showed promising durability *in vitro*, future studies should include longitudinal *in vivo* measurements. In particular, time-course analyses of background current and electrochemical performance are needed to assess signal integrity, biocompatibility, and electrode longevity over extended durations. In addition, applying an accelerated aging protocol under elevated temperature conditions may help predict long-term electrochemical stability and material degradation *in vitro*. Second, the current immunofluorescence analysis focused only on integrated density and basic cell counts. To better understand tissue responses, future studies should incorporate more multidimensional analyses. For example, depth-dependent neuron viability and spatial profiling of glial activation using high-resolution imaging techniques may provide richer, spatially resolved insights. Third, the electrode selectivity toward dopamine relative to other electroactive species, such as serotonin or glutamate, needs further exploration (Chang et al., 2012). Future work should involve selectivity testing under physiologically relevant interference conditions to evaluate the electrode’s suitability for complex neurochemical environments. Finally, studies in larger animal models are needed to evaluate their clinical translational potential. These models may help assess device stability, immune response, and targeting accuracy under conditions that better approximate human neuroanatomy.

Given its performance characteristics, the 30 µm cone-shaped CFME may also be applicable for detecting other neurochemicals beyond dopamine. Although this study did not measure serotonin directly, the electrode remained biocompatible and structurally stable for several days *in vivo*, as immunofluorescence shows. Zhao and Piatkevich (2023) reported that FSCV is widely used for serotonin monitoring but suffers from limited chemical selectivity and pronounced fouling due to serotonin oxidation products. While fouling was not evaluated here, our study’s stable signals and reduced immune response suggest a favorable electrode-tissue interface that could reduce signal degradation over time. These features support the potential use of the cone-shaped CFME for long-term serotonin sensing in regions such as the dorsal raphe nucleus, where reliable chronic measurements remain challenging.

Meunier et al. (2020) demonstrated that chronic implantation increases impedance and reduces capacitance in CFMEs due to biofouling, only partially reversible through electrochemical conditioning. Although this study did not include extended *in vivo* tracking, the consistent background current and oxidation responses observed acutely suggest that the sharpened geometry may reduce initial interface disruption. This observation aligns with Su et al., who emphasized that electrode geometry contributes to signal resolution and fouling resistance in chronic applications. While FSCV facilitates electrochemical surface renewal, the cone-shaped design may enhance this effect by minimizing interfacial degradation.

The stable and biocompatible properties of the cone-shaped CFME suggest its potential for integration into CMOS-based closed-loop neuromodulation platforms, as proposed by Rai et al. (2024), Rai et al. (2025). Such systems rely on robust electrochemical interfaces capable of delivering real-time feedback for therapeutic modulation. The demonstrated mechanical durability, stable signal performance, and minimal tissue disruption position the cone-shaped CFME as a viable front-end component for future lab-on-CMS platforms. Further efforts toward co-developing signal acquisition and processing circuits will be essential for realizing fully implantable, autonomous neurochemical monitoring systems.

## Conclusion

This study demonstrates that 30 µm cone-shaped CFMEs enhance *in vivo* dopamine detection by reducing tissue damage and extending electrode lifespan. Compared to conventional 7 μm and 30 µm bare CFMEs, the cone-shaped design minimized neuroinflammation, improved biocompatibility, and increased operational longevity by 4.7-fold, addressing key limitations in chronic neurochemical monitoring. These findings are consistent with previous reports identifying surface degradation (Meunier et al., 2020) and fouling susceptibility (Su et al., 2020) as major barriers to chronic electrode use. The cone-shaped geometry offers a structural solution by enhancing electrochemical resilience and preserving signal performance. Furthermore, the electrode’s stability and biocompatibility align with requirements for integration into CMOS-based neuromodulation platforms (Rai et al., 2024; Rai et al., 2025). These characteristics suggest potential use in closed-loop applications, including serotonin-sensitive adaptive DBS for neuropsychiatric disorders.

## Data Availability

The original contributions presented in the study are included in the article/Supplementary Material, further inquiries can be directed to the corresponding author.

## Abbreviations

CFME, carbon fiber microelectrode; DBS, deep brain stimulation; FSCV, fast scan cyclic voltammetry; IACUC, Institutional Animal Care and Use Committee; MFB, medial forebrain bundle; SEM, Scanning electron microscopy; EDS, Energy-dispersive X-ray spectroscopy; PBS, Phosphate-buffered saline.
