# Supplementary material for: Improved longevity and in vivo performance of neurotransmitter detection using 30 µm cone-shaped carbon fiber microelectrode
Source: Front Bioeng Biotechnol. 2025 Aug 22;13:1579380. doi: 10.3389/fbioe.2025.1579380 (PMC12411426; doi:10.3389/fbioe.2025.1579380)
Supplement: Supplementary file 1 [file DataSheet1.docx]

Supplementary Material

**Table of Contents**

**Figure S1 *In vitro* properties of 7 µm and 30 µm bare electrodes 3**

**Figure S2 EDS analysis of the 30 µm bare and cone-shaped CFMEs 3**

**Figure S3 Dopamine detection at different concentration 4**

**Figure S4 Background currents of 30 µm bare and cone-shaped CFMEs in striatum 4**


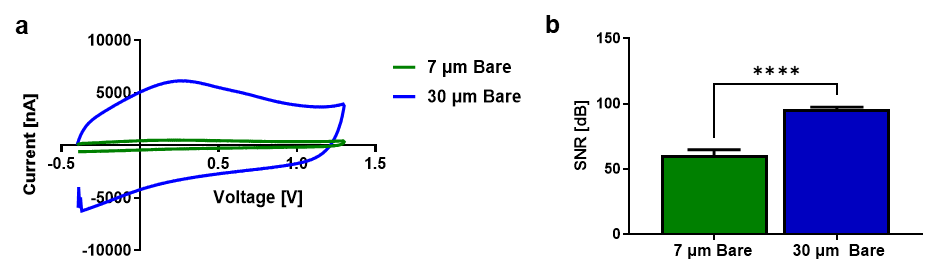


**Figure S1. Characteristics of 7 µm and 30 µm bare electrodes *in vitro*.**

(a) Voltammograms of background current in 1 µM DA solution. (b) Signal to noise ratio in decibel. SNR is calculated by dividing the signal amplitude for 1 µM DA by the average noise amplitude of 10 seconds preceding 3 seconds prior to the addition of DA. The green color represents 7 µm CFMEs and the blue color represents 30 µm CFMEs. Data represent mean ± SD. ****p<0.0001 by Welch’s t-test (n=5)


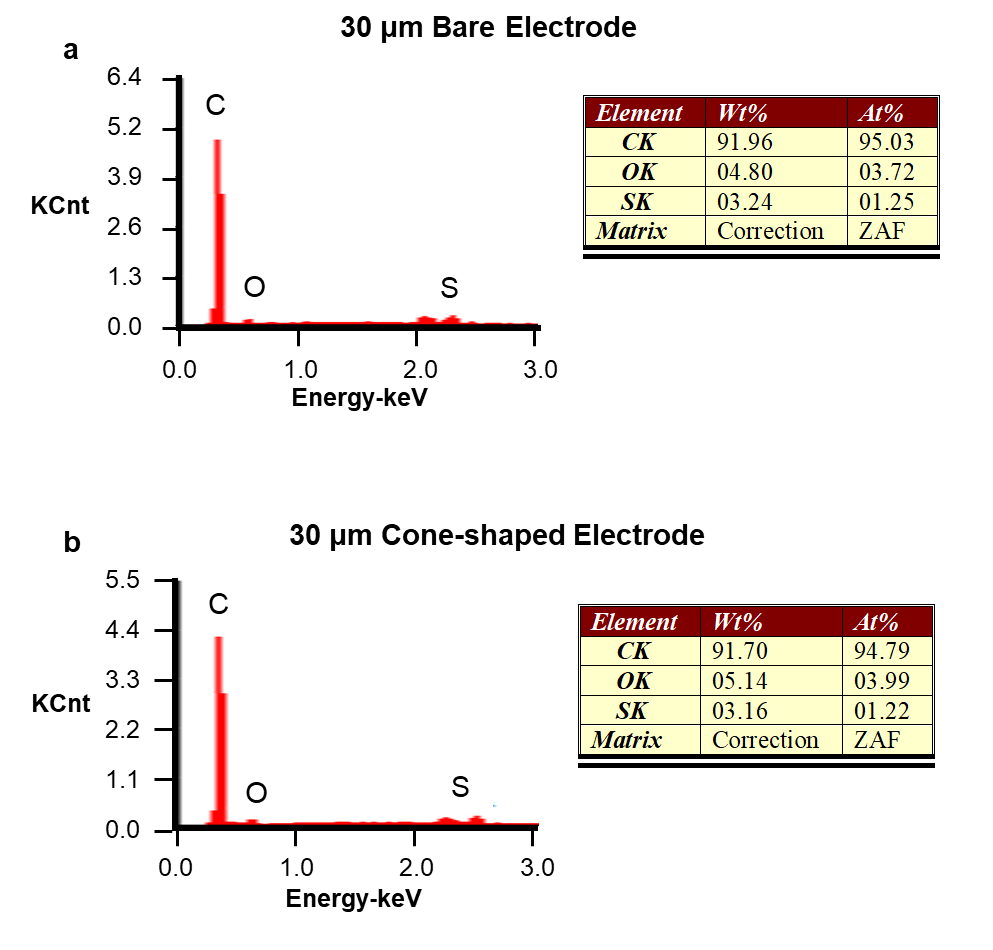


**Figure S2. EDS analysis of the 30 µm bare and cone-shaped electrodes.**

Representative EDS analysis results for 30 µm (a) bare and (b) cone-shaped carbon fiber microelectrodes.


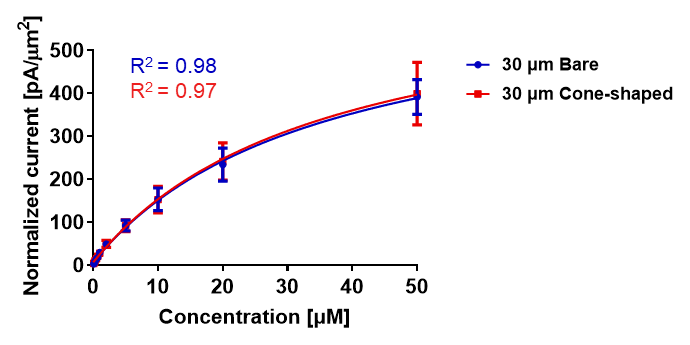


**Figure S3. Dopamine detection at different concentration.**

Dopamine detection at different concentration ranging from 100 nM to 50 µM (n=3). There is no significant differences (p=0.95).

**Figure S4. Background currents of 30 µm bare and cone-shaped CFMEs in striatum.**

Voltammograms of background current in striatum. The blue color represents 30 µm bare CFMEs and the red color represents 30 µm cone-shaped CFMEs.
